# Biotechnological Production of Poly(3-Hydroxybutyrate-*co*-4-Hydroxybutyrate-*co*-3-Hydroxyvalerate) Terpolymer by *Cupriavidus* sp. DSM 19379

**DOI:** 10.3390/bioengineering6030074

**Published:** 2019-08-26

**Authors:** Dan Kucera, Ivana Novackova, Iva Pernicova, Petr Sedlacek, Stanislav Obruca

**Affiliations:** 1 Faculty of Chemistry, Brno University of Technology, Purkynova 118, 612 00 Brno, Czech Republic; 2 Material Research Centre, Faculty of Chemistry, Brno University of Technology, Purkynova 118, 612 00 Brno, Czech Republic

**Keywords:** polyhydroxyalkanoates, terpolymer, P(3HB-*co*-3HV-*co*-4HB), *Cupriavidus malaysiensis*

## Abstract

The terpolymer of 3-hydroxybutyrate (3HB), 3-hydroxyvalerate (3HV), and 4-hydroxybutyrate (4HB) was produced employing *Cupriavidus* sp. DSM 19379. Growth in the presence of γ-butyrolactone, ε-caprolactone, 1,4-butanediol, and 1,6-hexanediol resulted in the synthesis of a polymer consisting of 3HB and 4HB monomers. Single and two-stage terpolymer production strategies were utilized to incorporate the 3HV subunit into the polymer structure. At the single-stage cultivation mode, γ-butyrolactone or 1,4-butanediol served as the primary substrate and propionic and valeric acid as the precursor of 3HV. In the two-stage production, glycerol was used in the growth phase, and precursors for the formation of the terpolymer in combination with the nitrogen limitation in the medium were used in the second phase. The aim of this work was to maximize the Polyhydroxyalkanoates (PHA) yields with a high proportion of 3HV and 4HB using different culture strategies. The obtained polymers contained 0–29 mol% of 3HV and 16–32 mol% of 4HB. Selected polymers were subjected to a material properties analysis such as differential scanning calorimetry (DSC), thermogravimetry, and size exclusion chromatography coupled with multi angle light scattering (SEC-MALS) for determination of the molecular weight. The number of polymers in the biomass, as well as the monomer composition of the polymer were determined by gas chromatography.

## 1. Introduction

Polyhydroxyalkanoates (PHA) represent a very attractive family of materials which are considered as an alternative to petrochemical polymers in applications which may benefit from their fully biodegradable and biocompatible nature. PHA are produced via fermentation since they are biosynthesized by numerous prokaryotes in the form of intracellular granules primarily as storage of carbon and energy [1]. Nevertheless, according to recent findings, PHA also plays a crucial role in the stress robustness and resistance of bacterial cells against various stress factors [2,3]. 

PHA are disadvantaged in competition with petrochemical polymers by their high-production cost. Since a substantial amount of the final cost is attributed to the cost of the carbon substrate, there are many attempts to produce PHA from inexpensive or even waste products in the food industry [4] such as waste lipids [5,6], crude glycerol formed as a side product of biodiesel production [7,8], various lignocellulose materials [9], or even carbon dioxide [10,11].

In general, the material properties of PHA strongly depend upon monomer composition. The homopolymer of 3-hydroxybutyrate (3HB), poly(3-hydroxybutyrate) (P3HB) is the most studied member of the PHA family, as it possesses numerous desirable properties. It is very interesting that the material in the native intracellular granules is completely amorphous and demonstrates extraordinary properties resembling super-cooled liquid [12]; nevertheless, when extracted from bacterial biomass, it quickly crystalizes. Therefore, its application potential is limited mainly by its high crystallinity, which reduces flexibility and elongation of the material. Nevertheless, the properties of the materials could be tuned when other monomer structures are incorporated into the polymer chain by feeding microbial culture with a suitable precursor(s). Therefore, copolymers containing, aside from 3HB, 3-hydroxyvalarate (3HV) subunits could be gained when microbial culture is cultivated in the presence of a suitable precursor with an odd number of carbon atoms such as propanol, propionate, pentanol, valerate, etc. The resulting copolymer poly(3-hydroxybutyrate-*co*-3-hydroxyvalerate) (P[3HB-*co*-3HV]) reveals substantially improved material properties and decreased crystallinity [13]. Similarly, some bacterial strains exposed to 1,4-butanediol or γ-butyrolactone (GBL) are able to biosynthesize copolymers containing 3HB and 4-hydroxybutyrate (4HB) monomer units. The copolymer poly(3-hydroxybutyrate-*co*-4-hydroxybutyrate) (P[3HB-*co*-4HB]) reveals mechanical properties, which resemble thermoplastic elastomers [14]. Moreover, PHA possessing 4HB subunits demonstrate increased biodegradability because lipases, which with PHA depolymerases, also have the ability to degrade P(3HB-*co*-4HB) [15], show higher activity at a higher fraction of 4HB [16]. Therefore, they find numerous high-value applications in the medical field [17]. Of course, terpolymer P(3HB-*co*-3HV-*co*-4HB) containing all of the above-mentioned monomer subunits demonstrate even superior properties and could be used in numerous fields and applications [18].

There are several reports dealing with the production of P(3HB-*co*-3HV-*co*-4HB) terpolymers employing various microorganisms. *Cupriavidus necator* (formerly *Alcaligenes eutrophus*, *Ralstonia eutropha* and *Wautersia eutropha*) was capable of desirable terpolymer production when cultivated on GBL and propionate; it was observed that propionate served not only as a 3HV precursor but it also increased the efficiency of 4HB incorporation into the terpolymer chain [19]. Similarly, Cavalheiro et al. produced P(3HB-*co*-3HV-*co*-4HB) by *Cupriavidus necator* using crude glycerol as the main carbon source, GBL as the 4HB precursor, and the 3HV-related precursor compound propionic acid [20]. Also, *Haloferax mediterranei* could be employed for the production of the terpolyester poly(3HB-*co*-3HV-*co*-4HB) without the need for a specific 3HV precursor which is based on the extraordinary metabolism of this microorganism, since it is capable of 3HV production from structurally unrelated carbon sources such as sugars or glycerol [21]. Finally, Ramachandran et al. used *Cupriavidus* sp. USMAA2-4 (now designated as *Cupriavidus malaysiensis* DSM 19379) for the terpolymer production from oleic acid and various 4HB and 3HV precursors [22].

In this work, we attempted to develop an efficient process of P(3HB-*co*-3HV-*co*-4HB) production employing *Cupriavidus malaysiensis* DSM 19379. We aimed at the maximization of both PHA yields, as well as 3HV and 4HB monomer fractions in the polymer to achieve desired material properties of the produced materials. Various culture strategies were used for this purpose.

## 2. Materials and Methods 

### 2.1. Microorganisms and Cultivation

*Cupriavidus malaysiensis* USMAA2-4 (DSM 19379) was purchased from Leibnitz Institute DSMZ-German Collection of Microorganism and Cell Cultures, Braunschweig, Germany. The nutrient broth (Himedi—10 g/L Peptone, 10 g/L Beef Extract, 5 g/L NaCl) (NB) medium was used for the inoculum development. The mineral salt medium (MSM) for cultivation was composed of 3 g/L (NH_4_)_2_SO_4_, 1.02 g/L KH_2_PO_4_, 11.1 g/L Na_2_HPO_4_ · 12 H_2_O, 0.2 g/L MgSO_4_ · 7 H_2_O, and 1 mL/L of microelement solution, the composition of which was as follows: 9.7 g/L FeCl_3_ ∙ 6 H_2_O, 7.8 g/L CaCl_2_ ∙ 2 H_2_O, 0.156 g/L CuSO_4_ ∙ 5 H_2_O, 0.119 g/L CoCl_2_ ∙ 2 H_2_O, 0.118 g/L NiCl_2_ ∙ 4 H_2_O, and 1 L 0.1 M HCl. The following carbon sources were used to prepare the production media: GBL (8 g/L) (Sigma Aldrich, Steinheim, Germany); ε-caprolactone (8 g/L) (Sigma Aldrich, Steinheim, Germany); 1,4-butanediol (8 g/L) (Sigma Aldrich, Schnelldorf, Germany); 1,6-hexanediol (8 g/L) (Sigma Aldrich, Schnelldorf, Germany); fructose (20 g/L); glucose (20 g/L); sunflower oil (20 g/L); glycerol p.a. (20 g/L) (Lach-ner, Neratovice, Czechia). Carbon sources, salt solutions, and microelement solutions were autoclaved separately (121 °C, 20 min) and then aseptically reconstituted at room temperature prior to the inoculation (inoculum ratio was 10 vol%). The cultivations were performed in Erlenmeyer flasks (volume 250 mL) containing 100 mL of MSM. The temperature was set at 30 °C, the agitation at 180 rpm. The cells were harvested after 72 h of cultivation as described in Section 2.2. For a successful centrifugation process, the medium was heated to 70 °C for 15 min.

#### 2.1.1. Single-Stage Cultivation Mode

GBL or 1,4-butanediol were used to prepare the production media in the same way as described in Section 2.1. Propionic acid (Sigma Aldrich, Schnelldorf, Germany) and valeric acid (Sigma Aldrich, Schnelldorf, Germany) as 3HV precursors were added at a concentration 1 g/L to media after 24 h of cultivation to minimize their toxic effect on growth of the microbial culture. After another 48 h of cultivation, the cells were harvested. The total length of the cultivation was 72 h. As a control, we chose to cultivate without adding any of the precursors of 3HV.

#### 2.1.2. Two-Stage Cultivation Mode

Glycerol (20 g/L) or combination of glycerol and 1,4-butanediol (12 and 8 g/L, respectively) were used to prepare the production media based on MSM. After 48 h of cultivation (30 °C, 180 rpm), biomass was separated by centrifugation (6000 rpm, 4 °C) and aseptically transferred to fresh MSM with 0.1 g/L (NH_4_)_2_SO_4_, 8 g/L 1,4-butanediol and 1 g/L valeric acid. Cultivation without valeric acid served as a control. The cells were harvested after another 48 h of cultivation (30 °C, 180 rpm).

### 2.2. Determination of the CDM and PHA Content

To determine the biomass concentration and PHA content in cells, samples (10 mL) were centrifuged (6000 rpm) and then the cells were washed with distilled water. The biomass concentration expressed as cell dry mass (CDM) was analyzed as reported previously [23]. The PHA content of dried cells was analyzed by gas chromatography (GC) (Trace GC Ultra, Thermo Scientific, Waltham, MA, USA) as reported by Brandl et al. [24]. Commercially available P(3HB-*co*-3HV) (Sigma Aldrich, Schnelldorf, Germany) composed of 88 mol% 3HB and 12 mol% 3HV were used as a standard and benzoic acid (LachNer, Neratovice, Czechia) was used as an internal standard. In addition to the quantification of total PHA in biomass, GC was also used to determine the monomeric composition and to determine the molar content of individual monomers in the obtained polymers.

### 2.3. Polymer Characterization

Following four polymers obtained by *Cupriavidus malaysiensis*, USMAA2-4 (DSM 19379) using different substrates and cultivation strategies were selected due to various 4HB content for polymer characterization: Sample 1—single-stage, fructose (20 g/L); Sample 2—single-stage, 1,4-butanediol + valeric acid; Sample 3—single-stage, 1,4-butanediol + propionic acid; Sample 4—two-stage, glycerol (20 g/L), and then 1,4-butanediol + valeric acid.

To determine the molecular weight of PHA, approximately 20 mg CDM was washed in 5 mL chloroform at 70 °C for 24 h under continuous stirring. Solid residues were separated by filtration and, finally, the solvent was removed by evaporation at 70 °C to a constant weight. The obtained polymer was also used for DSC analysis. After that, 5 mg of the polymer was solubilized in 1 mL of HPLC-grade chloroform and passed through syringe filters (nylon membrane, pore size 0.45 μm). Samples were analyzed by gel Size Exclusion Chromatography (Agilent, Infinity 1260 system containing PLgel MIXED-C column) coupled with Multiangle Light Scattering (Wyatt Technology, Dawn Heleos II, Goleta, CA, USA) and Differential Refractive Index (Wyatt Technology, Optilab T-rEX, Goleta, CA, USA) detection [24]. The weight-average molecular weight (Mw) and polydispersity index (Đ) were determined using the ASTRA software (Wyatt Technology, Goleta, CA, USA) based on Zimm´s equations.

Melting behavior of the isolated PHA polymers was analyzed by means of a differential scanning calorimeter (DSC) Q2000 (TA Instruments, New Castle, DE, USA) equipped with an RCS90 cooling accessory as previously described by Kucera et al. [25]. Phase transitions of mercury and indium were used for the calibration in the applied temperature range. Approximately 5 mg of sample was placed in hermetically sealed Tzero aluminum pans, and the measurement was carried out under a dynamic nitrogen atmosphere. To ensure the same thermal history of all samples prior to the evaluation of their melting behavior, each sample was first heated at 10 °C/min to 190 °C and subsequently cooled down to −30 °C at the same cooling rate. Then the sample was heated again (10 °C/min to 200 °C) and the thermogram, recorded in this second heating step, was further evaluated.

Thermogravimetric analysis of the isolated polymers was performed on Q5000 TGA analyzer (TA Instruments, New Castle, DE, USA). During the analysis, a known weight of a sample (ca 5 um) was heated at 10 °C/min to 800 °C under oxidative atmosphere (air). The major decomposition step, characterized by a rapid fall in the sample weight in the temperature range 250 °C to 350 °C, was further processed using TGA data evaluation software Universal Analysis 2000 (TA Instruments, New Castle, DE, USA). The automated evaluation of the weight change provided two characteristic temperatures of the degradation step: onset temperature of the thermal decomposition (Td_onset_) and temperature corresponding to the maximal rate of the weight change (Td_max_).

## 3. Results and Discussion

### 3.1. Biosynthesis of P(3HB-co-4HB) Copolymer 

*Cupriavidus malaysiensis* DSM 19379 was employed to produce polyhydroxyalkanoates (PHA) using different carbon sources. This bacterium was isolated from water samples collected from Sg. Pinang river, Penang, Malaysia based on its ability to produce various types of PHA, including copolymers containing 4HB [26]. According to our results shown in Table 1, P3HB or P(3HB-*co*-4HB) were produced according to the type of the substrate. The bacterial strain was capable to produce copolymer P(3HB-*co*-4HB) only in the presence of precursors structurally related to 4HB such as GBL, 1,4-butandiol, ε-caprolactone, or 1,6-hexanediol e.g., diols and carboxylic acids possessing hydroxy group at last carbon atom which is agreement with results of Rahayu et al. [27] The highest PHA titers were achieved when four-carbon precursors of 4HB such as 1,4-butanediol or GBL were used. When such a structural motif was lacking, the strain accumulated homopolymer consisting exclusively of 3HB subunits. In the results, the strain appears to be unable to utilize oil because the CDM yield was low, and GC did not reveal PHA in the cell structure. There is a significant difference between utilization of fructose and glucose. While the yield of CDM and PHA with fructose was 10.78 g/L and 7.54 g/L, respectively, the yield with glucose was only 2.29 g/L CDM and 0.23 g/L PHA. This is not a very surprising result, also the closely related wild-type strain *Cupriavidus necator* H16 is not able to efficiently utilize glucose because it does not possess the activity of 6-phosphofructokinase [28]. *Cupriavidus malaysiensis* USMAA2-4 was also able to utilize glycerol reaching relatively high biomass titers; nevertheless, PHA production was the lowest among the substrates used which enabled PHA biosynthesis.

### 3.2. Biosynthesis of P(3HB-co-3HV-co-4HB) Terpolymer through Single-Stage Cultivation

The following experiments were focused on the production of the terpolymer P(3HB-*co*-3HV-*co*-4HB). To obtain the desired material, 1,4-butanediol and GBL have been selected as carbon sources since the bacteria can utilize these substances for growth but also incorporate them into the copolymer P(3HB-*co*-4HB). Sodium propionate and valeric acid were tested in this experiment as odd carbon atom precursors for the synthesis of 3HV monomer incorporated into the terpolymer chain. Results of the single-stage terpolymer production including yields of CDM and PHA are shown in Table 2.

In the resulting Table 2, CDM column shows that it generally achieved better growth using 1,4-butanediol as carbon sources than with GBL. Surprisingly, with the addition of the precursors for terpolymer synthesis, the CDM gain was higher. Valeric acid appears to be superior in the production of the P(3HB-*co*-4HB-*co*-3HV) terpolymer. With the addition of this precursor, significant growth was achieved with both GBL and 1,4-butanediol. The highest biomass concentration was obtained using 1,4-butanediol in combination with valeric acid, with a biomass yield of 8.68 g/L.

The highest PHA production was achieved in combination with valeric acid. The PHA yields were 0.82 g/L and 1.79 g/L for GBL and 1,4-butanediol, respectively. Thus, the combination of 1,4-butanediol with valeric acid again appears to be the best for production terpolymer in the single-stage strategy. Regarding the composition of the polymers obtained in this experiment, the terpolymer was synthesized using both precursors of 3HV. However, a higher 3HV fraction was obtained using valeric acid. In the case of propionate, generation of 3HV requires activity of 3-ketothiolase coupling propionyl-CoA and acetyl-CoA such as BktB in *C. necator* H16 [29]. On the contrary, conversion of valerate into 3HV could be relatively simply performed within the first “turn” of β-oxidation. It is likely that *Cupriavidus* DSM 19379 reveals relatively lower 3-ketohiolase activity as compared to the activity of the β-oxidation pathway, and therefore, valerate seems to be superior to the 3HV precursor for terpolymer synthesis as compared with propionate. In the case of terpolymer composition, the highest 3HV content was achieved using GBL together with valeric acid. The monomeric composition of the P(3HB-*co*-3HV-*co*-4HB) terpolymer was 56.22, 17.92, and 25.85 mol%, respectively. The polymer produced by *Cupriavidus malaysiensis* DSM 19379 using 1,4-butanediol in combination with valeric acid had almost the same composition. Nevertheless, it should be pointed out that the overall PHA productivity gained in the single-stage process was relatively low. The PHA content was about 20 weight percent of CDM and gained PHA titers were, therefore, also low. Hence, we attempted to improve the productivity of the culture by employing the two-stage cultivation.

### 3.3. Biosynthesis of the P(3HB-co-3HV-co-4HB)Tterpolymer through the Two-Stage Cultivation

To enhance PHA productivity, we performed an additional experiment in which cultivation was performed in two steps. In the first step, we aimed at a cultivation of maximal biomass using glycerol (20 g/L) as a cheap carbon source. According to our results, glycerol stimulates growth of the bacterium, but it is not converted into P(3HB) which could be taken as an advantage since the production of a desirable terpolymer with low 3HB fraction could be achieved in the second step. In addition, glycerol (12 g/L) was also mixed with 1,4-butanediol (8 g/L) in a parallel series of cultivations. The second stage was performed in the cultivation media with nitrogen limitation and 1,4-butanediol, and most importantly, 1,4-butanediol and valeric acids were used as 4HB and 3HV precursors, respectively. Valeric acid was chosen as the precursor of the 3HV since it was identified as the superior 3HV precursor for the investigated culture. The first phase of cultivation served to obtain a high amount of PHA-poor biomass. PHA production was then achieved by nitrogen limitation in the second phase. All results are shown in Table 3.

From the results of this experiment shown above, it could be seen that the bacterial strain grew best when, in the first step, glycerol was used in combination with 1,4-butanediol and in the second one, 1,4-butanediol with valeric acid. The biomass gain was 5.94 g/L. Conversely, the smallest growth was achieved by cultivation using glycerol followed by 1,4-butanediol, where only 1.60 g/L CDM was obtained. CDM and PHA analysis was also performed on cultures after the first stage of the two-stage production. The assumption that at this stage a biomass with a low PHA content would be obtained, has been fulfilled. The glycerol-based medium reached 3.1 g/L CDM containing 5.3% PHB. Using a substrate containing 14-BD, we obtained 3.8 g/L CDM containing 24.0% P(3HB-*co*-4HB).

From the results, *Cupriavidus malaysiensis* DSM 19379 can efficiently synthesize the desired terpolymer P(3HB-*co*-3HV-*co*-4HB), PHA contents in bacterial cells are substantially higher when two-stage cultivation strategy was adopted. The highest weight fraction, 69.64 wt%, as well as the highest PHA gain, 4.14 g/L, was achieved when glycerol was used together with 1,4-butanediol in the first step and 1,4-butanediol with valeric acid in the second. Regarding polymer composition, good results were achieved when 1,4-butanediol was used in combination with valeric acid in the second step. When only glycerol was used in the first step, we obtained a terpolymer composed of 53.78 mol% 3HB, 16.76 mol% 4HB, and 29.46 mol% 3HV. Using glycerol together with 1,4-butanediol in the first step, a terpolymer composed of 3HB 65.68 mol%, 4HB 16.46 mol%, 3HV 17.86 mol% was subsequently obtained. It seems that a combination of glycerol and 1,4-butanediol in the first step of cultivation and 1,4-butanediol and valeric acid in the second stage of the cultivation is a very promising strategy which results in very high PHA titers and high PHA content in the cells and also high portions of 4HB and 3HV in the terpolymer structure.

### 3.4. Characteristics of Isolated Polymers

Differential scanning calorimetry and thermogravimetry were chosen to study the thermal properties of the polymers; size exclusion chromatography was used to determine molecular weight and polydispersity index of the polymers. The following samples of isolated polymers were selected for analysis. Sample No. 1 is a control polymer containing almost exclusively 3HB monomer units. Sample No. 2 was collected by cultivation using a combination of 1,4-butanediol and valeric acid; the proportion of 3-hydroxyvalerate in this sample is 14.65 mol%. Sample No. 3 was obtained from cultivation using 1,4-butanediol and sodium propionate, and the concentration of 3HV was 8.32 mol%. The last sample was isolated from a cell suspension-cultured to produce a terpolymer, using glycerol followed by 1,4-butanediol together with valeric acid. In this sample, the 3HV molar ratio was highest at all, namely 29.46 mol%. The results are placed in Table 4. From the thermograms recorded by differential scanning calorimetry, we determined glass transition temperature (Tg) and melting point (Tm). The total heat of fusion ΔH, was also determined via integration of the melting endotherm. Using thermogravimetry, the degradation onset temperature (Td_onset_) and the temperature that corresponds to the maximal rate of sample decomposition (Td_max_) were determined.

**Figure 1:**
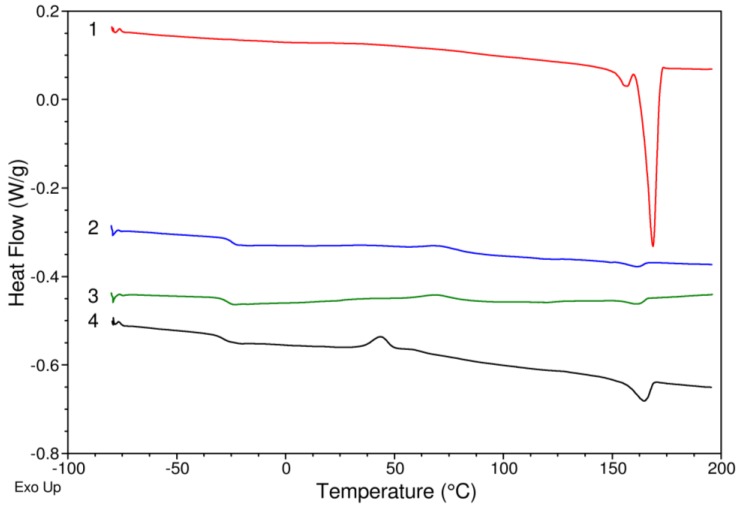
Results of DSC analysis of isolated polymers.

Comparison of DSC thermograms of the four isolated polymers is shown in Figure 1. In Sample No. 1, there is a sharp melting endotherm which appears at about 170 °C, which is typical of polyhydroxybutyrate. The peak area corresponds to the heat released in this process. The large area of the melting endotherm indicates a high tendency of the polymer to crystallize spontaneously which in turn causes no significant signs of glass transition and cold crystallization are found in its thermogram as compared to the other three analyzed samples. Further, the sample is characterized by a double peak at the melting point, indicating that the polymer crystallites are present in two forms with distinct thermal stability. On the other hand, for all the terpolymer samples (Samples 2–4), it can be seen at first sight that much less intensive melting peak is shown on the curves. Furthermore, apparent glass transition and cold crystallization of the polymer chains altogether indicates significantly reduced the tendency for spontaneous crystallization. In other words, involvement of and additional monomer to the copolymer structure resulted in a more amorphous structure. Incorporating 3HV into the polymer structure also caused a decrease in melting point to about 161 °C. Fahima Azira et al. [30] produced terpolymer P(3HB-co-3HV-co-4HB) and the melting points ranged from 160 to 164 °C.

The SEC-MALS technique was used to measure weight average molecular weight (Mw) of obtained polymers and values ranged from 137 to 314 kDa. The highest value was measured for the sample with the highest molar ratio of 4HB and the lowest for the sample with the highest molar ratio of 3HV. The Mw values measured are typical for the bacterial strain used and consistent with other studies [22].

Thermogravimetric analysis was performed in order to compare the thermal stability of the produced polymers. In a respective thermogram, decomposition of a polymer is represented by the onset temperature of the decomposition (the temperature at which the polymer starts to decompose, Td_onset_) and by the temperature which corresponds to the maximal rate of the decomposition (Td_max_). Among the isolated P(3HB-*co*-3HV-*co*-4HB) terpolymer samples, the highest degradation temperature (i.e., the highest thermal stability of the polymer) was measured for Sample No. 3 composed of 63.81 mol% 3HB, 27.87 mol% 4HB, and 8.32 mol% 3HV. This is the sample with the lowest 3HV but the highest 4HB. This suggests that a higher proportion of 4HB in the terpolymer leads to the higher thermal stability of the polymer. Thus, sample 3 has the most promising properties from a technological point of view because its melting point was set at near lowest, 161.67 °C, and the degradation temperature to highest 300.83 °C. The wide temperature window between melting temperature and degradation temperature is important for polymer processing. When working with the melt, it is important that it does not decompose.

## 4. Conclusions

To sum-up, in flask experiments, we have developed a two-stage cultivation strategy which is based on the application of glycerol and 1,4-butanediol as the carbon substrates in the first stage of cultivation, after that the cells are transferred into nitrogen-limited cultivation media with 1,4-butanediol and valeric acids. This cultivation strategy provides high PHA yields and PHA content in bacterial cells. Moreover, the P(3HB-co-3HV-co-4HB) terpolymer with low 3HB fraction and high 3HV and 4HB contents is obtained. The material properties of obtained polymers were consistent with materials produced in previous studies aimed at the production of P(3HB-co-3HV-co-4HB) terpolymers. In our future experiments, we will transfer the process into laboratory bioreactors to evaluate its suitability for industrial production of PHA. 

## Figures and Tables

**Table 1:** Substrates for P3HB and P(3HB-*co*-4HB) production by *Cupriavidus* sp. DSM 19379.

Substrate	CDM (g/L)	PHA (wt%)	PHA (g/L)	4HB (mol%)	3HB (mol%)
fructose	10.78 ± 0.06	69.95 ± 0.42	7.54 ± 0.10	0	100
glucose	2.29 ± 0.06	10.03 ± 0.06	0.23 ± 0.02	0	100
glycerol	4.60 ± 0.04	5.30 ± 0.05	0.24 ± 0.02	0	100
sunflower oil	1.33 ± 0.05	0	0	0	0
GBL	4.50 ± 0.02	35.84 ± 0.92	1.61 ± 0.12	22.18 ± 1.06	77.82 ± 1.06
1,4-butanediol	4.01 ± 0.02	11.67 ± 0.06	0.47 ± 0.03	23.12 ± 1.61	76.88 ± 1.61
ε-caprolactone	0.22 ± 0.04	42.80 ± 0.61	0.10 ± 0.04	68.89 ± 1.12	31.11 ± 1.12
1,6-hexanediol	2.64 ± 0.01	39.83 ± 0.95	1.05 ± 0.07	34.35 ± 0.96	65.65 ± 0.96

**Table 2:** Single stage terpolymer production (72 h cultivation, application of 3HV precursor at the 24 h of cultivation 1 g/L).

Primary Substrate	3HV Precursor	CDM (g/L)	PHA (g/L)	PHA (wt%)	3HB (mol%)	4HB (mol%)	3HV (mol%)
GBL	none	3.64 ± 0.03	0.81 ± 0.05	22.14 ± 0.01	68.40 ± 0.23	31.60 ± 0.23	0
	propionic acid	5.06 ± 0.37	0.62 ± 0.06	12.16 ± 0.00	69.18 ± 0.22	23.41 ± 0.05	7.41 ± 0.16
	valeric acid	7.97 ± 1.85	0.82 ± 0.09	10.41 ± 0.01	56.22 ± 0.32	25.85 ± 0.40	17.92 ± 0.07
1,4-butanediol	none	7.41 ± 0.51	1.05 ± 0.19	14.44 ± 0.02	68.97 ± 2.26	31.03 ± 2.26	0
	propionic acid	8.19 ± 0.35	1.65 ± 0.43	20.01 ± 0.04	63.81 ± 1.71	27.87 ± 0.10	8.32 ± 1.80
	valeric acid	8.68 ± 0.14	1.79 ± 0.88	20.52 ± 0.10	60.63 ± 2.90	24.72 ± 7.42	14.65 ± 4.53

**Table 3:** Two-stage terpolymer production (48 h at glycerol or glycerol + 1,4-butanediol, after that transfer to nitrogen-limited medium with precursor of 3HV.

Primary Substrate	Secondary Precursor	CDM (g/L)	PHA (g/L)	PHA (wt%)	3HB (mol%)	4HB (mol%)	3HV (mol%)
Glycerol	1,4-butanediol	1.60 ± 0.03	0.84 ± 0.02	52.25 ± 0.12	80.85 ± 0.68	18.09 ± 0.26	1.06 ± 0.43
	1,4-butanediol + valeric acid	2.73 ± 0.58	1.42 ± 0.25	52.12 ± 1.76	53.78 ± 0.61	16.76 ± 0.87	29.46 ± 0.26
Glycerol + 1,4-butanediol	1,4-butanediol	3.26 ± 0.11	2.09 ± 0.01	64.14 ± 2.38	77.89 ± 0.53	21.60 ± 0.54	0.51 ± 0.01
	1,4-butanediol + valeric acid	5.94 ± 0.14	4.14 ± 0.05	69.64 ± 0.73	65.68 ± 1.02	16.46 ± 1.28	17.86 ± 0.26

**Table 4:** Properties of the selected materials.

Sample	3HB (mol%)	4HB (mol%)	3HV (mol%)	Mw (kDa)	Đ (-)	Tg (°C)	Tm (°C)	ΔH (J/g)	Td_onset_ (°C)	Td_max_ (°C)
1	99.33	0.67	0	155.97	1.04	-	155.79	4.70	271.88	287.94
							168.70	64.89		
2	60.63	24.72	14.65	258.66	1.02	24.78	161.34	2.80	271.48	293.49
3	63.81	27.87	8.32	314.60	1.01	26.19	161.67	3.04	275.24	300.83
4	53.78	16.76	29.46	137.89	1.17	29.00	164.63	12.69	271.36	295.53

## References

[1] Kourmentza C., Plácido J., Venetsaneas N., Burniol-Figols A., Varrone C., Gavala H.N., Reis M.A. (2017). Recent advances and challenges towards sustainable polyhydroxyalkanoate (PHA) production. Bioengineering.

[2] Obruca S., Sedlacek P., Koller M., Kucera D., Pernicova I. (2018). Involvement of polyhydroxyalkanoates in stress resistance of microbial cells: Biotechnological consequences and applications. Biotechnol. Adv..

[3] Slaninova E., Sedlacek P., Mravec F., Mullerova L., Samek O., Koller M., Hesko O., Kucera D., Marova I., Obruca S. (2018). Light scattering on PHA granules protects bacterial cells against the harmful effects of UV radiation. Appl. Microbiol. Biotechnol..

[4] Haas C., Steinwandter V., De Apodaca E.D., Madurga B.M., Smerilli M., Dietrich T., Neureiter M. (2015). Production of PHB from chicory roots - Comparison of three *Cupriavidus necator* strains. Chem. Biochem. Eng. Q..

[5] Verlinden R.A.J., Hill D.J., Kenward M.A., Williams C.D., Piotrowska-Seget Z., Radecka I.K. (2011). Production of polyhydroxyalkanoates from waste frying oil by *Cupriavidus necator*. Amb Express.

[6] Ciesielski S., Mozejko J., Pisutpaisal N. (2015). Plant oils as promising substrates for polyhydroxyalkanoates production. J. Clean. Prod..

[7] Jiang G., Hill D.J., Kowalczuk M., Johnston B., Adamus G., Irorere V., Radecka I. (2016). Carbon sources for polyhydroxyalkanoates and an integrated biorefinery. Int. J. Mol. Sci..

[8] Moita R., Freches A., Lemos P.C. (2014). Crude glycerol as feedstock for polyhydroxyalkanoates production by mixed microbial cultures. Water Res..

[9] Obruca S., Benesova P., Marsalek L., Marova I. (2015). Use of lignocellulosic materials for PHA production. Chem. Biochem. Eng. Q..

[10] Meixner K., Kovalcik A., Sykacek E., Gruber-Brunhumer M., Zeilinger W., Markl K., Haas C., Fritz I., Mundigler N., Stelzer F. (2018). Cyanobacteria Biorefinery—Production of poly(3-hydroxybutyrate) with *Synechocystis salina* and utilisation of residual biomass. J. Biotechnol..

[11] Troschl C., Meixner K., Drosg B. (2017). Cyanobacterial PHA Production—Review of Recent Advances and a Summary of Three Years’ Working Experience Running a Pilot Plant. Bioengineering.

[12] Sedlacek P., Slaninova E., Enev V., Koller M., Nebesarova J., Marova I., Hrubanova K., Krzyzanek V., Samek O., Obruca S. (2019). What keeps polyhydroxyalkanoates in bacterial cells amorphous? A derivation from stress exposure experiments. Appl. Microbiol. Biotechnol..

[13] Koller M. (2018). Chemical and biochemical engineering approaches in manufacturing polyhydroxyalkanoate (PHA) biopolyesters of tailored structure with focus on the diversity of building blocks. Chem. Biochem. Eng. Q..

[14] Lee W.H., Azizan M.N.M., Sudesh K. (2004). Effects of culture conditions on the composition of poly(3-hydroxybutyrate-*co*-4-hydroxybutyrate) synthesized by *Comamonas acidovorans*. Polym Degrad Stab..

[15] Rodríguez-Contreras A., Calafell-Monfort M., Marqués-Calvo M.S. (2012). Enzymatic degradation of poly(3-hydroxybutyrate-*co*-4-hydroxybutyrate) by commercial lipases. Polym. Degrad. Stabil..

[16] Saito Y., Nakamura S., Hiramitsu M., Doi Y. (1996). Microbial synthesis and properties of poly(3-hydroxybutyrate-*co*-4-hydroxybutyrate). Polym. Int..

[17] Singh A.K., Srivastava J.K., Chandel A.K., Sharma L., Mallick N., Singh S.P. (2019). Biomedical applications of microbially engineered polyhydroxyalkanoates: an insight into recent advances, bottlenecks, and solutions. Appl. Microbiol. Biotechnol..

[18] Chanprateep S., Kulpreecha S. (2006). Production and characterization of biodegradable terpolymer poly(3-hydroxybutyrate-*co*-3-hydroxyvalerate-*co*-4-hydroxybutyrate) by *Alcaligenes* sp. A-04. J. Biosci. Bioeng..

[19] Lee Y.H., Kang M.S., Jung Y.M. (2000). Regulating the molar fraction of 4-hydroxybutyrate in poly(3-hydroxybutyrate-4-hydroxybutyrate) biosynthesis by *Ralstonia eutropha* using propionate as a stimulator. J. Biosci. Bioeng..

[20] Cavalheiro J.M., Raposo R.S., de Almeida M.C.M., Cesário M.T., Sevrin C., Grandfils C., Da Fonseca M.M.R. (2012). Effect of cultivation parameters on the production of poly(3-hydroxybutyrate-*co*-4-hydroxybutyrate) and poly(3-hydroxybutyrate-4-hydroxybutyrate-3-hydroxyvalerate) by *Cupriavidus necator* using waste glycerol. Biores. Technol..

[21] Hermann-Krauss C., Koller M., Muhr A., Fasl H., Stelzer F., Braunegg G. (2013). Archaeal production of polyhydroxyalkanoate (PHA) co-and terpolyesters from biodiesel industry-derived by-products. Archaea.

[22] Ramachandran H., Iqbal N.M., Sipaut C.S., Abdullah A.A.A. (2011). Biosynthesis and characterization of poly (3-hydroxybutyrate-*co*-3-hydroxyvalerate-*co*-4-hydroxybutyrate). Terpolymer with various monomer compositions by *Cupriavidus* sp. USMAA2-4. Appl. Biochem. Biotechnol..

[23] Obruca S., Marova I., Melusova S., Mravcova L. (2011). Production of polyhydroxyalkanoates from cheese whey employing *Bacillus megaterium* CCM 2037. Ann. Microbiol..

[24] Brandl H., Gross R.A., Lenz R.W., Fuller R.C. (1988). *Pseudomonas oleovorans* as a source of poly(beta-hydroxyalkanoates) for potential application as a biodegradable polyester. Appl. Environ. Microb..

[25] Kucera D., Pernicová I., Kovalcik A., Koller M., Mullerova L., Sedlacek P., Mravec F., Nebesarova J., Kalina M., Marova I. (2018). Characterization of the promising poly(3-hydroxybutyrate) producing halophilic bacterium *Halomonas halophila*. Biores. Technol..

[26] Amirul A.A., Yahya A.R.M., Sudesh K., Azizan M.N.M., Majid M.I.A. (2008). Biosynthesis of poly(3-hydroxybutyrate-*co*-4-hydroxybutyrate) copolymer by *Cupriavidus* sp. USMAA1020 isolated from Lake Kulim, Malaysia. Biores. Technol..

[27] Rahayu A., Zaleha Z., Yahya A.R.M., Majid M.I.A., Amirul A. (2008). A Production of copolymer poly(3-hydroxybutyrate-co-4-hydroxybutyrate) through a one-step cultivation process. World J. Microbiol. Biotechnol..

[28] Lopar M., Špoljarić I.V., Cepanec N., Koller M., Braunegg G., Horvat P. (2014). Study of metabolic network of *Cupriavidus necator* DSM 545 growing on glycerol by applying elementary flux modes and yield space analysis. J. Ind. Microbiol. Biotechnol..

[29] Lindenkamp N., Peplinski K., Volodina E., Ehrenreich A., Steinbuchel A. (2010). Impact of multiple beta-ketothiolase deletion mutations in *Ralstonia eutropha* H16 on the composition of 3-mercaptopropionic acid-containing copolymers. Appl. Environ. Microbiol..

[30] Fahima Azira T.M., Nursolehah A.A., Norhayati Y., Majid M.I.A., Amirul A.A. (2011). Biosynthesis of Poly(3-hydroxybutyrate-co-3-hydroxyvalerate-co-4-hydroxybutyrate) terpolymer by *Cupriavidus* sp. USMAA2-4 through two-step cultivation process. World J. Microbiol. Biotechnol..

